# *De novo* sequencing and comparative transcriptome analysis of adventitious root development induced by exogenous indole-3-butyric acid in cuttings of tetraploid black locust

**DOI:** 10.1186/s12864-017-3554-4

**Published:** 2017-02-16

**Authors:** Jine Quan, Seng Meng, Erhui Guo, Sheng Zhang, Zhong Zhao, Xitian Yang

**Affiliations:** 1 The Environment and Ecology Key Laboratory of of Education Ministry in West China, Northwest A&F University, Taicheng Road 3, Yangling, Shaanxi 712100 China; 2 Department of Forestry, Henan Agricultural University, Zhengzhou, Henan Province China

**Keywords:** de novo, Transcriptome, Adventitious root development, IBA, Tetraploid black locust

## Abstract

**Background:**

Indole-3-butyric acid (IBA) is applied to the cuttings of various plant species to induce formation of adventitious roots (ARs) in commercial settings. Tetraploid black locust is an attractive ornamental tree that is drought resistant, sand tolerant, can prevent sand erosion and has various commercial uses. To further elucidate the mechanisms of AR formation, we used Illumina sequencing to analyze transcriptome dynamics and differential gene expression at four developmental stages in control (CK) and IBA-treated groups.

**Results:**

The short reads were assembled into 127,038 unitranscripts and 101,209 unigenes, with average lengths of 986 and 852 bp. In total, 10,181 and 14,924 differentially expressed genes (DEGs) were detected in the CK and IBA-treated groups, respectively. Comparison of the four consecutive developmental stages showed that 282 and 260 DEGs were shared between IBA-treated and CK, suggesting that IBA treatment increased the number of DEGs. We observed 1,721 up-regulated and 849 down-regulated genes in CI vs. II, 849 up-regulated and 836 down-regulated genes in CC vs. IC, 881 up-regulated and 631 down-regulated genes in CRP vs. IRP, and 5,626 up-regulated and 4,932 down-regulated genes in CAR vs. IAR, of which 25 up-regulated DEGs were common to four pairs, and these DEGs were significantly up-regulated at AR. These results suggest that substantial changes in gene expression are associated with adventitious rooting. GO functional category analysis indicated that IBA significantly up- or down-regulated processes associated with regulation of transcription, transcription of DNA dependent, integral to membrane and ATP binding during the development process. KEGG pathway enrichment indicated that glycolysis/gluconeogenesis, cysteine and methionine metabolism, photosynthesis, nucleotide sugar metabolism, and lysosome were the pathways most highly regulated by IBA. We identified a number of differentially regulated unigenes, including 12 methionine-related genes and 12 ethylene-related genes, associated with the KEGG pathway cysteine and methionine metabolism. The GO enrichment, pathway mapping, and gene expression profile analyses revealed molecular traits for root induction and initiation.

**Conclusion:**

Our study presents a global view of the transcriptomic profiles of tetraploid black locust cuttings in response to IBA treatment and provides new insights into the fundamental mechanisms associated with auxin-induced adventitious rooting.

**Electronic supplementary material:**

The online version of this article (doi:10.1186/s12864-017-3554-4) contains supplementary material, which is available to authorized users.

## Background

Tetraploid black locust (*Robinia pseudoacacia L*.) is an attractive ornamental tree that has various commercial uses. It exhibits fast growth, drought resistance, saline-alkaline tolerance and low soil fertility requirements, and it is the primary tree species used as a wind break and for sand fixation and for soil and water conservation in the Loess Plateau Region [1]. Tetraploid black locust does not root well and thus is difficult to plant in a variety of environments. However, the phytohormone auxin can promote the formation of adventitious roots (ARs) in cuttings [1, 2]. The formation of ARs relies on the method used. In agricultural practice, plant loss is usually caused by AR formation and slow-rooting cuttings. Therefore, it is believed that the formation of ARs is necessary for the smooth spreading of cuttings of tetraploid black locust.

AR formation is a highly complex regenerative process that is influenced by numerous internal and external factors, including environmental conditions, phytohormones and nutritional status [3–6]. Auxin is a crucial phytohormone that promotes AR formation in cuttings [7]. However, the mechanisms underlying the role of auxin in AR formation are only superficially understood, and the lack of details at the molecular level limits improvements to cutting propagation.

The auxin IBA is widely used in woody plant propagation to induce rooting. Although indole-3-acetic acid (IAA) is the primary native auxin in plants, IBA is more effective in promoting ARs [8–11]. For example, treatment of cuttings with IBA significantly improves rooting rates in *Terminalia tomentosa* [8], *Pinus contorta* [12, 13], *Malus pumila* [14, 15], and *Pinus radiate* [16]. Brinker et al. [17] reported that IBA induces the expression of genes involved in cell replication and cell-wall weakening but inhibits genes related to auxin transport, photosynthesis and cell-wall synthesis during *P. contorta* root initiation. Thus, the processes that occur in cuttings after IBA treatment, and particularly the functions of IBA-regulated genes, should be elucidated. These results also indicate that IBA may directly or indirectly induce the formation and differentiation of root primordia. We previously demonstrated that 5.4 mmol/L IBA significantly increases the rooting rate of tetraploid black locust hardwood cuttings to approximately 80% [1, 2], in contrast to approximately 2% AR formation in CK cuttings. To explore the significant impact of IBA on rooting, our recent studies have primarily focused on tetraploid black locust at the anatomical, physiological and biochemical levels [1, 2]. Developments in molecular biology and proteomics techniques have allowed us to investigate IBA-induced AR development in tetraploid black locust via homology cloning, quantitative real-time PCR (qPCR), two-dimensional electrophoresis and protein bio-mass spectrometry (MALDI-TOF, Q-TOF), resulting in the isolation and identification of hundreds of IBA-response-related genes and proteins [18–20]. The studies cited above represent the first explorations of genes involved in AR formation in tetraploid black locust, but transcriptomic information and identification of many genes related to IBA-induced AR development is scarce. The molecular mechanism of rooting in tetraploid black locust is complex, and the mechanism by which IBA promotes the formation of ARs in cuttings remains unclear due to the lack of transcriptomic and genomic information. Therefore, our study represents a necessary acceleration of the acquisition of transcriptomes related to IBA-induced AR development in tetraploid black locust cuttings. Transcriptomic studies of IBA-induced AR development have been conducted in *Camellia sinensis* [21], *Petunia hybrid* [22], *Pinus contorta* [23], and *Morus alba L.* [24]. These studies primarily focused on stem cuttings and involved techniques such as Illumina sequencing [21], massively parallel signature sequencing [17], EST analysis [23], microarray analysis, and suppression subtraction hybridization [11]. However, the transcriptome of tetraploid black locust has not been studied yet.

The rapid development of next-generation sequencing (NGS) technology has improved the efficiency and reduced the cost of Illumina/Solexa sequencing technology. The results of Illumina/Solexa sequencing are highly reproducible, both technically and biologically [23, 25]. Illumina/Solexa sequencing technology is the most widely used NGS technology for the *de novo* sequencing and analysis of the transcriptomes of non-model organisms.

In this study, we used the Illumina sequencing platform to analyze CK and IBA-treated cuttings of tetraploid black locust in order to identify new genes involved in the IBA-induced formation of ARs in cuttings and to obtain deeper insight into the mechanism of propagation in tetraploid black locust. The application of Illumina next-generation sequencing provides more transcripts to facilitate further genomic studies of tetraploid black locust. This study presents the transcriptome for IBA-treated cuttings and provides a genetic resource for improving woody plant propagation.

## Results

### IBA induced adventitious root formation in tetraploid locust

**Fig. 1:**
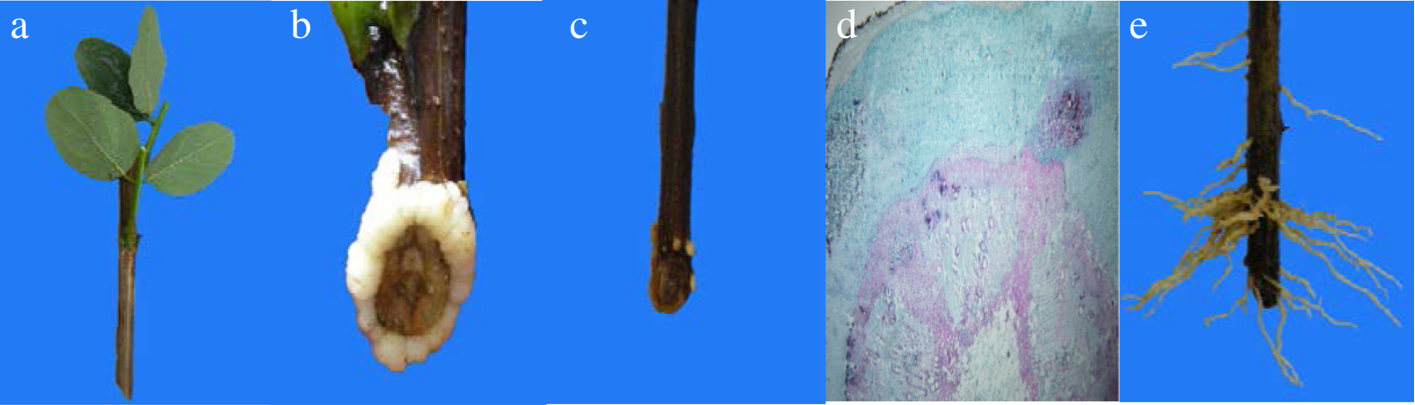
Morphological changes in tetraploid *black* locust cuttings undergoing adventitious root development in a sand bed. **a** Softwood cuttings before cutting. **b** *White* callus appeared 10 days after cutting. **c**-**d** *Yellow* callus appeared and tiny adventitious roots emerged (root primordium) at 15 days after cutting. **e** Adventitious root formation and elongation at 20 days after cutting. As biological replicates, 10 samples were randomly selected from the groups treated with IBA

We found that approximately 80% of the softwood cuttings form roots after treatment with the optimal concentration of IBA. The process of AR formation in the softwood cuttings involves the following steps (Fig. 1): First, softwood cuttings after IBA treatment via soaking were inserted into seeding beds (Fig. 1a). During the 7–10 days after IBA treatment, we observed that white calli had formed on the wound surfaces of the soft cuttings (Fig. 1b). During the 15–20 days after the cuttings were treated, tiny AR primordia (RP) formed and subsequently developed into root meristems (Fig. 1c and d). During the last stage, the ARs on the cuttings were formed and elongated (Fig. 1e).

### Illumina sequencing, *de novo* assembly and sequence analysis

To identify genes involved in IBA-induced AR formation, we subjected cDNA preparations from the basal parts of IBA-treated or CK cuttings to *de novo* sequencing on the Illumina HiSeq 2000 platform. In total, eight cDNA preparations were sequenced from control cuttings sampled at the first stage (CI), white callus stage (CC), primordia formation stage (CRP), and AR formation stage (CAR), as well as from IBA-treated cuttings sampled at the first stage (II), white callus stage (IC), primordia formation stage (IRP), and AR formation stage (IAR). The total number of raw reads produced for each library ranged from 33.88 million to 46.99 million. The raw data have been submitted to the NCBI repository (https://www.ncbi.nlm.nih.gov/geo/). After filtering, the number of high-quality clean reads per library ranged from 33.74 million to 46.81 million, and the Valid Ratio (Reads) % ranged from 98.96 to 100.00% (Table 1). The short reads were assembled into 127,038 unitranscripts and 101,209 unigenes with average lengths of 986 and 852 bp, total lengths of 125,353,356 and 86,239,985 bp, and N50 lengths of 1,643 and 1,449 bp, respectively (Additional file 1). The number of reads per kilobase of exonic sequence per million of total reads sequenced (RPKM) was used to calculate the transcript abundance in each sample. The average RPKM ranged from 7.41 to 10.44 in CK group and from 7.72 to 9.49 in the IBA treatment group (Table 1). These results indicate that overall transcript abundance was greatly increased in both the CK and IBA-treated groups over the course of AR development. Moreover, the average RPKM of the IBA treatment group was greater than that of the CK at the first stage, white callus stage and primordial formation stage, reflecting a marked increase in gene transcription produced by IBA treatment.

**Table 1:** Summary for RNA-Seq data of tetraploid black locust

Sample	Raw reads	Clean reads	Valid ratio%	Average RPKM
CI	41709122	41709122	100.00	7.41
II	41834616	41834616	100.00	7.72
CC	43327202	42946218	99.12	7.54
IC	43228082	42776422	98.96	7.92
CRP	42564996	42389290	99.59	7.78
IRP	46993652	46818876	99.63	8.73
CAR	37674292	37668220	99.98	10.44
IAR	33882086	33747400	99.60	9.49

**Fig. 2:**
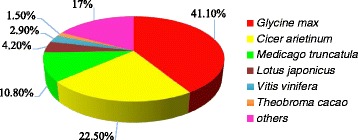
The species distribution of unigene blastx results against the NCBI-Nr protein database

The unigenes were aligned to five public protein databases (Nr, KOGs, KEGG, Pfam, and Swiss-Prot). The majority of the unigenes were annotated using the Nr (74.36%) database (Additional file 2). These results indicate that the Nr database is an informative platform for the functional annotation of tetraploid black locust. Additionally, the Nr database queries revealed that the highest percentage of tetraploid black locust sequences most closely matched sequences from *Glycine max* (41.1%), followed by *Cicer arietinum* (22.5%), *Medicago truncatula* (10.8%), *Lotus japonicus* (4.2%), *Vitis vinifera* (2.9%) and *Theobroma cacao* (1.5%) (Fig. 2). The results indicated that overall homology was highest to model legume plants, implying that the sequences of the tetraploid black locust transcripts obtained were assembled and annotated properly in this study [26].

### Transcriptome changes during AR development in tetraploid black locust cuttings

**Fig. 3:**
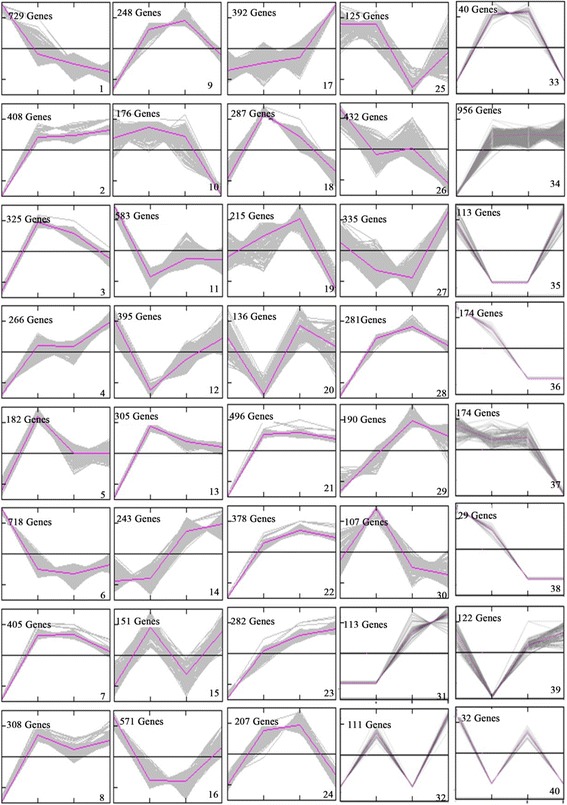
RNA-seq-based transcriptome dynamics of IBA-treated cuttings during adventitious root development. The fold-change >2.0 for each gene was used for the hierarchical clustering analysis at each of the four selected developmental stages (II, IC, IRP and IAR). The 14,924 genes were classified into 40 regulation patterns (groups 1–10, 14–18, and 20–24)

In this study, we used IBA-treated cuttings as a model in which to investigate transcriptome changes during AR development and formation. In total, 10,181 and 14,924 DEGs were detected in at least one of the four stages in the CK and IBA-treated groups, respectively. The transcriptomic changes in the cuttings during AR development were examined by cluster analysis of gene expression patterns, which categorized the 14,924 identified genes into 40 groups (Fig. 3); 1,854 genes expressed in three or fewer stages belonged to groups 31 to 40. The largest group (group 34) contained 956 genes whose expression decreased continuously over the course of AR development. The expression levels of the 282 genes in group 23 increased continuously over the course of the four developmental stages. Group 23 included genes encoding an ethylene-responsive transcription factor, an auxin-induced protein and a zinc finger protein. The 113 genes in group 35 were not expressed at stage II or stage IC. The cluster analysis also revealed that the abundances of 89.7% of the transcripts detected in the IBA-treated cuttings varied over the course of AR development (Fig. 3). Comparison of the expression patterns of IBA-treated (Fig. 3) and CK (Additional file 3) cuttings revealed that the 40 groups were common to both the IBA-treated and CK cuttings, and the expression patterns of 97.5% of the genes expressed in CK were similar to those of the genes expressed in the IBA-treated cuttings (the exceptions belonged to groups 17 and 23).

Moreover, the expression levels of 282 DEGs (group 23) in the IBA-treated cuttings and of 260 DEGs (group 5) in CK increased continuously. The expression of 61 DEGs increased continuously in both IBA-treated and CK cuttings (Additional file 4). These results suggest that a substantial alteration of gene expression is associated with adventitious rooting. For example, the expression of the beta HLH protein 93 (Trb7468921), early-responsive to dehydration stress protein (ERD4) (Trb4932701), ethylene responsive element binding factor 1 (Trb5562722), peroxidase 2 (Trb4932701), and zinc-finger protein 1 (Trb8690271) DEGs increased continuously in the IBA-treated cuttings. In addition, the expression of the C2H2-like zinc finger protein (Trb5430271), cold, circadian rhythm, and RNA binding 1 (Trb6574322), embryo-specific protein 3 (Trb4689243), and heat shock protein 60 (Trb7645302) DEGs increased continuously in the CK cuttings. Moreover, the expression of the ACC oxidase 1 (Trb7020711), basic helix-loop-helix (bHLH) DNA-binding protein 5 (Trb8657204), cell wall/vacuolar inhibitor of fructosidase 2 (Trb5894705), LOB domain-containing protein 41 (Trb6654701), and CAP160 protein (Trb3457711) DEGs increased in both IBA-treated and CK cuttings.

### DEGs in IBA effects on adventitious root development

**Fig. 4:**
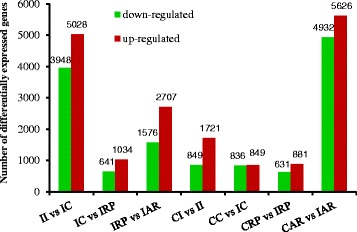
Differentially expressed genes among stages in IBA treatment and between IBA-treated and CK for each developmental stage

Genes that were differentially expressed in the four developmental stages were identified using IDEG6. Genes were determined to be IBA-regulated if they had fold-change > 2 and *P* ≤ 0.05 in at least one rooting stage. In total, 14,924 DEGs were observed in the four developmental stages of the IBA-treated cuttings. The genes that exhibited differences in expression between two consecutive rooting stages are shown in Fig. 4. A comparison of tetraploid black locust cuttings in stage II and stage IC revealed that 8,976 genes were differentially expressed, of which 3,948 were down-regulated and 5,028 were up-regulated in stage IC (Fig. 4). Genes showing significant differential expression included 3 zinc finger domain-containing proteins (Trb5503102, Trb5503104, Trb5503101), 2 unknown proteins (Trb7570401, Trb2435501), embryonic abundant protein USP92 (Trb7595601), squamosa promoter-binding-like protein 13-like (Trb4932701), PEBP family protein (Trb7547281), and 4 auxin-induced proteins (Trb6569103, Trb5697601, Trb5697602, Trb5476601). A total of 1,677 DEGs were differentially expressed between stage IC and stage IRP, of which 643 were down-regulated and 1,034 were up-regulated in stage IRP. Genes showing significant differential expression included 2 hypothetical proteins (Trb5933302, Trb5933301), nodulin-26 (Trb14811401), bidirectional sugar transporter SWEET3-like (Trb6691501), aquaporin TIP1-1 (Trb2089901), a peptide/nitrate transporter (Trb6088201), a mitochondrial alternative oxidase (Trb6400801), momilactone A synthase-like (Trb5040301), sugar transport protein 13-like, zeatin O-glucosyltransferase-like, and UDP-glycosyltransferase 74B1-like (Trb4128302, Trb3408601, Trb6028401). A total of 4,383 genes were differentially expressed between the IRP stage and the final stage (IAR), of which 1,576 were down-regulated and 2,707 were up-regulated in stage IAR. Genes showing significant differential expression included 2 protease inhibitor-like proteins (Trb5017202 and Trb5017201), 4 disease resistance proteins (Trb2657401, Trb7602201, Trb4619901, Trb4619902), 6 chlorophyll a-b binding protein CP26, chloroplastic-like isoforms (Trb56455601, Trb6596903, Trb6596905, Trb6596902, Trb5674201, Trb6596911), S-adenosylmethionine decarboxylase proenzyme belonging in spermine biosynthesis (Trb4357111, Trb4357101, Trb4357121), S-adenosylmethionine synthase and methionine synthase in the S-adenosylmethionine biosynthetic process (Trb48279501, Trb80561501, Trb865153011), 1-aminocyclopropane-1-carboxylate synthase in the ethylene biosynthetic process (Trb34955101), auxin response factor 18-like (Trb20505701), and 6 abscisic acid receptor PYL6-like proteins (Trb6836312, Trb6836315, Trb6653601, Trb6653602, Trb6836313, Trb6833901). Moreover, 6,877, 549, and 2,557 genes showed specific regulation only between stages II and IC, IC and IRP, and IRP and IAR, respectively (Additional file 5). Additionally, 581 DEGs were common between stages II vs. IC and stages IC vs. IRP, 178 DEGs were common between stages IC vs. IRP and stages IRP vs. IAR, and 1,151 DEGs were common to stages II and IC and stages IRP and IAR (Additional file 5). The comparison of the four consecutive developmental stages showed that 368 DEGs were common to all four stages. Among them, ten and six genes were continuously up-regulated and down-regulated, respectively, in all four developmental stages. These genes are mainly involved in methionine metabolism pathways. Several highly induced genes, such as ACC oxidase (Trb7020711), a zinc finger family protein (Trb6191801) and a wound-responsive protein (Trb74240111) were expressed most highly during the stages IRP and IAR (Additional file 5). The functional classification of the differentially expressed unigenes in the four developmental stages is shown in Additional file 6. These results indicate that these genes may promote rooting in tetraploid black locust cuttings. The DEGs included more up-regulated transcripts than down-regulated transcripts, indicating that many genes responded positively to IBA treatment. This result is consistent with previous studies in Arabidopsis and tomato [27–29].

### DEGs in response to IBA during the four developmental stages of adventitious rooting

Analysis using the Z-score method suggested that in each of the four developmental stages, the expression levels of 16,325 genes differed significantly between the transcriptomes of IBA and CK at *P* < 0.05 and fold-change > 2. Of these, 2570 unigenes, 1721 up-regulated and 849 down-regulated, were differentially expressed in CI vs. II; 1,685 unigenes, 849 up-regulated and 836 down-regulated, were differentially expressed in CC vs. IC; 1,512 unigenes, 881 up-regulated and 631 down-regulated, were differentially expressed in CRP vs. IRP; and 10,558 unigenes, 5,626 up-regulated and 4,932 down-regulated, were differentially expressed in CAR vs. IAR (Fig. 4). In addition, 10,588 genes (64.86% of the total) showed differential expression between CK and IBA treatment only during the last stage. The identification of more differentially expressed genes in the final stage might be related to the greater distinction of this developmental stage. Moreover, in all four developmental stages, there were more up-regulated genes than down-regulated genes. These results suggest that IBA treatment increased the number of genes that were up-regulated to promote adventitious rooting.

Only 25 up-regulated DEGs were detected in all four stages (Additional file 7). These included seven individual genes, namely, ACC oxidase 1 (ACO1, Trb70207), arabinogalactan protein 22 (AGP22, Trb44105), flavodoxin-like quinone reductase 1 (FQR1, Trb50725), multidrug resistance-associated protein 5 (AtMRP5, Trb74407), photosystem II light harvesting complex gene 2 (LHCB2, Trb78279), response regulator 9 (ARR9, Trb73071) and uclacyanin 3 (UCC3, Trb51917), and ten gene families (13 genes), namely, an ARM repeat superfamily protein (Trb61444), an auxin-responsive family protein (Trb63108), a bifunctional inhibitor/lipid-transfer protein/seed storage 2S albumin super family protein (Trb55916), a C2H2-type zinc finger family protein (Trb25048), an NmrA-like negative transcriptional regulator family protein (Trb76075), a P-loop-containing nucleoside triphosphate hydrolase super family protein (Trb68969), a rhodanese/cell cycle control phosphatase super family proteins (Tr73938), two thiamine pyrophosphate-dependent pyruvate decarboxylase family protein (Trb64980 and Trb66324), three wound-responsive family proteins (Trb51890, Trb68418 and Trb51890) and a WRKY family transcription factor (Trb62764), as well as five proteins of unknown function (Trb69581, Trb64606, Trb43851, Trb73653 and Trb55166). These genes likely play an important role in the development of adventitious rooting in response to IBA treatment [30].

Additionally, several DEGs encode auxin-related products, such as indole-3-acetic acid synthetase (Trb7222702) and serine/threonine-protein kinase protein (Trb7444204). Six of these DEGs encode a1-aminocyclopropane-1-carboxylate oxidase (Trb7020714), which was previously reported to be involved in ethylene biosynthesis [31].

### GO enrichment analysis

**Fig. 5:**
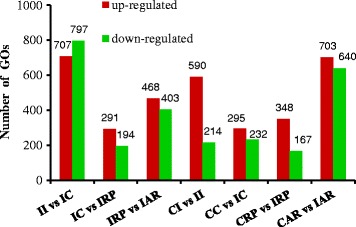
The distribution of GO terms enriched in the sample pairs

To discern global patterns of differential transcript abundance over the time course, unigenes with contrasting significance at *P* ≤0.05 were further filtered to include only those with values greater than fold-change > 2 in a comparison of unigene abundance between the samples [32]. For the groups of up-regulated and down-regulated genes, we applied WEGO to compare the GO classifications of these genes [33]. Comparing the IBA-treated cuttings to the CK cuttings at each of the four developmental stages, the results showed significantly more up-regulated GO classifications than down-regulated GO classifications at all developmental stages (Fig. 5). Further, when comparing pairs of consecutive rooting stages of the IBA-treated cuttings, there were significantly more up-regulated GO classifications than down-regulated GO classifications. These results indicate that in all four developmental stages, IBA-treated cuttings showed significant up-regulation of genes in a wide variety of GO classifications.

The GO analysis of the DEGs in the four developmental stages between IBA-treated and CK revealed that most of the encoded products were associated with the following GO categories: regulation of transcription, transcription of DNA dependent, integral to membrane and ATP binding. The most common categories associated with the AR stage were integral to membrane and ATP binding activity (Additional file 8). Several genes showed highly significant differences, including cytokinin dehydrogenase (Trb5994202), 1-aminocyclopropane-1-carboxylate oxidase (Trb7341803), 4-coumarate: CoA ligase-like 9 (Trb6655606), ubiquitin protein ligase RGLG1-like (Trb5586102), S-adenosylmethionine synthase (Trb5508801 and Trb9018001), spermidine synthase, S-adenosylmethionine decarboxylase (Trb6300002 and Trb4357121) and zeaxanthin epoxidase (Trb7243506). These auxin-responsive genes have important functions in AR formation (Additional file 9). In addition, highly significant differences in gene expression were detected, most of which were distributed among the following GO terms: cytokines in metabolic process, ethylene biosynthetic process, jasmonic acid biosynthetic process, auxin metabolic process, S-adenosylmethionine biosynthetic process, spermidine biosynthetic process and abscisic acid biosynthetic process.

The top 50 most significantly up- and down-regulated GO categories during the IBA-treated development process are listed in Additional file 10. The results revealed that the GO classifications associated with organism development, such as AR development, xylem development, phloem development, post-embryonic root development, and organ development, were significantly up-regulated in CI compared with II but significantly down-regulated at stage IRP compared with IAR. Hormone-related pathways, such as ethylene-mediated signaling pathway and auxin metabolic process, were significantly up-regulated in CI compared with II, whereas genes with the GO classifications abscisic acid biosynthetic process, jasmonic acid biosynthetic process and cytokinin metabolic process were significantly up-regulated at stage IRP compared with IAR.

### KEGG pathway enrichment analysis

To further determine which biological pathways were significantly (*P* ≤ 0.05) modulated during AR formation, KEGG pathway enrichment was performed using the KEGG Automatic Annotation Server (KAAS) [34] to reveal KEGG pathway enrichment in the transcriptomes of IBA-treated and CK cuttings at four developmental stages. Between stages CI and II, thirteen KOs were significantly down-regulated or up-regulated, including glycolysis/gluconeogenesis, cysteine and methionine metabolism, photosynthesis, amino sugar and nucleotide sugar metabolism, lysosome, and starch and sucrose metabolism. Between stages CC and IC, five KOs were significantly down-regulated or up-regulated, including alanine, aspartate and glutamate metabolism, bacterial secretion system, butanoate metabolism, and starch and sucrose metabolism. Further, when comparing stages CRP and IRP, seven KOs were significantly down-regulated or up-regulated, including Alanine, aspartate and glutamate metabolism, beta-Alanine metabolism, and Type I diabetes mellitus. Meanwhile, comparing stages CAR and IAR, twenty-five KOs were significantly down-regulated or up-regulated, including glycolysis/gluconeogenesis, carbon fixation in photosynthetic organisms, alzheimer’s disease, starch and sucrose metabolism, cysteine and methionine metabolism, arginine and proline metabolism, MAPK signaling pathway, ABC transporters, valine, leucine and isoleucine degradation, antigen processing and presentation, nitrogen metabolism, tryptophan metabolism and selenoamino acid metabolism (Additional file 11). These results suggest that significant metabolic changes occur during the period of AR formation.

To gain insight into the differential KOs specifically induced in cuttings by IBA at the four developmental stages, the pairs of consecutive stages in the IBA-treated group were compared: II vs. IC, IC vs. IRP, and IRP vs. IAR. Forty KOs were significantly down-regulated or up-regulated between stage II and stage IC, including glycolysis/gluconeogenesis, carbon fixation in photosynthetic organisms, pentose phosphate pathway, pyruvate metabolism, amino sugar and nucleotide sugar metabolism, cysteine and methionine metabolism, and alanine aspartate and glutamate metabolism. These results indicate that glycolysis/gluconeogenesis and carbon fixation in photosynthetic organisms were significantly up-regulated by IBA treatment in stage IC relative to stage II. In stage IRP compared with stage IC, the KO Phenylalanine metabolism was significantly down-regulated, and the group Starch and sucrose metabolism was significantly up-regulated. Phenylpropanoids contribute to plant defenses as inducible chemical barriers or as signaling molecules [35–37]. From stage IRP to stage IAR, fifteen KOs were significantly down-regulated or up-regulated, including alanine, aspartate and glutamate metabolism, carbon fixation in photosynthetic organisms, glycolysis/gluconeogenesis, arginine and proline metabolism, cysteine and methionine metabolism, pentose phosphate pathway, oxidative phosphorylation, and photosynthesis. cysteine and methionine metabolism, as well as ethylene pathway and associated polyamines, might play important roles in IBA-induced adventitious rooting [38]. Furthermore, the results further suggest that IBA increased the number of KOs and genes exhibiting changes in expression during the early stages of rooting.

### Verification of DEGs during the four developmental stages of adventitious rooting

**Fig. 6:**
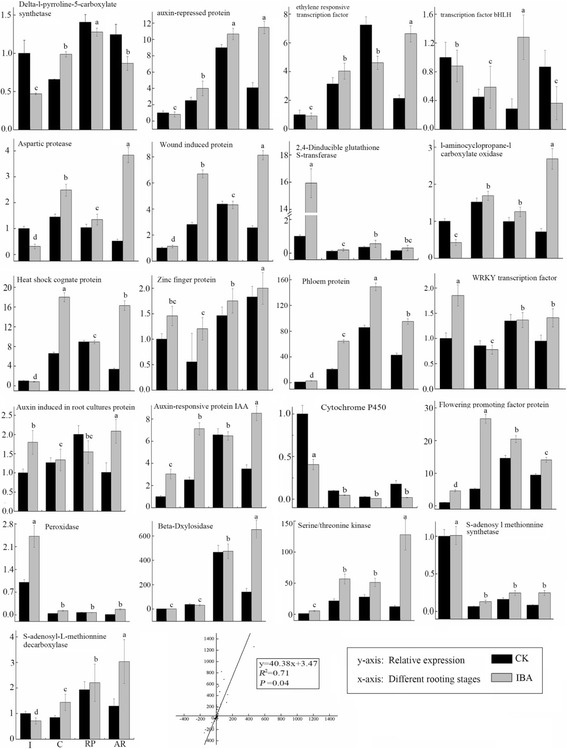
q-PCR validation of differential expression. Transcript levels of 21 genes in CK (*black column*) and IBA (*gray column*). The y-axis shows the relative gene expression levels as analyzed by q-PCR. Bars represent the standard error (*n* = 3). A Comparison of the gene expression ratios obtained from RNA-seq data and q-PCR

RNA was extracted from IBA-treated and CK cuttings at the four selected stages of AR development and used as the template for q-PCR-based validation of the sequence-based transcription profiles of 21 differentially expressed candidate unigenes. Detailed information on these genes is presented in Additional file 12. The selected genes were associated with methionine metabolism, plant hormone signal transduction, phenylalanine metabolism and other enzymatic processes (Fig. 6). Linear regression [(RNA-seq value) = a(q-PCR value) + b] analysis revealed an overall correlation coefficient of 0.71 according to Villacorta-Martín et al. and Yu et al. [39, 40]. The q-PCR analysis confirmed that the RNA-seq approach provided reliable data regarding differential gene expression during the AR developmental stages of tetraploid black locust cuttings.

The genes threonine kinase (Trb6720901), auxin-repressed protein (Trb2435601), ethylene responsive transcription factor (Trb6253801), auxin-induced in root cultures protein (Trb6310802), auxin-responsive protein IAA (Trb8029901), and SAMDC (Trb7651101) beta-D-xylosidase (Trb6237301) remained highly expressed during AR development for all cutting stages, and the expression of these genes was higher in IBA-treated cuttings than in CK cuttings in all four stages. We observed the highest expression of flowering promoting factor protein (Trb5566601) in the callus induction phase of IBA-treated cuttings, as well as high expression in the AR formation phase, and the expression of this gene in the AR formation phase was higher in IBA-treated cuttings than in CK cuttings.

We observed the highest expression levels of heat shock cognate protein (Trb5873209), zinc finger protein (Trb6191801), ACO (Trb7020711), heat shock cognate protein (Trb5873209), SAMS (Trb6301201) wound-induced protein (Trb7602201), and aspartic protease in guard cell (Trb7014201) in the AR formation phase of IBA-treated cuttings, and the expression of these genes in the AR formation phase was higher in IBA-treated cuttings than in CK cuttings. The most highly expressed protein detected was phloem protein (Trb6463501) in the root primordia formation phase of IBA-treated cuttings, as well as high expression in the AR formation phase, and expression of this gene in the AR formation phase was higher in IBA-treated cuttings than in CK cuttings. We observed the highest expression levels of Phloem WRKY transcription factor (Trb6276401), 2,4-D inducible glutathione S-transferase (Trb5981501), and peroxidase (Trb6791601) during the initiation formation phase of IBA-treated cuttings, as well as high expression in the AR formation phase, and expression of these genes in the AR formation phase was higher in IBA-treated cuttings than in CK cuttings. These results demonstrate that IBA might directly or indirectly regulate the expression of the above genes during AR development in tetraploid black locust. Several members of the threonine kinase, auxin-repressed protein, ethylene responsive transcription factor, auxin induced in root cultures protein, auxin-responsive protein IAA, ACO and SAMDC beta-D-xylosidase families have been identified and shown to mediate adventitious rooting [22, 41]. However, during the AR formation phase, the expression levels of the genes cytochrome p450 (Trb5844701), Delta-l-pyrroline-5-carboxylate synthetase (Trb6734101), and transcription factor bHLH (Trb6501202) were higher in CK cuttings than in IBA-treated cuttings. These results show that these genes were less affected by IBA treatment during the stages of AR development. Instead, cytochrome p450 and transcription factor bHLH genes are involved in responses to stress, such as drought and high salinity, thus leading to efficient adventitious rooting [11].

## Discussion

### Unigene determination of transcriptome sequencing during AR development in tetraploid black locust cuttings

Illumina RNA-seq technology has been extensively used for model plant transcriptome sequencing [42] with reference genome data or for non-model plants [43] without reference genomic information. In this study, the Illumina HiSeq 2000 platform was used to perform a *de novo* transcriptome sequencing analysis of the tetraploid black locust cuttings to better understand the gene expression changes during adventitious rooting. Pooled RNA samples from IBA-treated and CK cuttings sampled at four time points after AR excision were used to construct cDNA libraries for deep sequencing. In this sequencing, approximately 33.74 million to 46.81 million paired-end clean reads were obtained from the IBA-treated and CK cuttings at the four time points. After *de novo* assembly, we obtained 101,209 unigenes with a mean length of 986 bp, which is longer than has been reported previously in studies using the same technology [26, 28, 43, 44].

We identified a total of 10,181 and 14,924 DEGs (fold change > 2) were detected in at least one of the four stages in the CK and IBA treated cuttings, respectively. The genes that exhibited differences in expression between two consecutive rooting stages are shown in Fig. 4. A comparison of tetraploid black locust cuttings in stage II and stage IC revealed that 8,976 genes were differentially expressed, of which 3,948 were down-regulated and 5,028 were up-regulated in stage IC. Using a DNA microarray method, Rigal et al. [30] studied gene expression changes during adventitious rooting in the model tree *Populus trichocarpa*. Their results indicated that 5,781 genes were differentially expressed in the organization of the AR primordium; 6,538 genes were differentially expressed during primordium differentiation; and 1,146 genes were differentially expressed between these two stages [9]. In another similar study using cDNA microarrays, Brinker et al. [17] identified 220 genes whose expression changed significantly during root development in hypocotyl cuttings of *Pinus contorta* [6]. The results obtained suggest that RNA-Seq is a sensitive, low-cost, and accurate method for deep-sequencing the transcriptomes of plant without available genomic information, and this method was able to identify more DEGs during the early stages of adventitious rooting relative to the results of DNA microarrays. This technology also enables the precise elucidation of transcripts in the samples.

The associations between the GO terms and the lists of DEGs were investigated using GO functional enrichment analysis. Significantly enriched GO terms for the DEGs in the II vs. CI, IC vs. CC, IRP vs. CRP and IAR vs. CAR comparisons included cellular component, cell part, membrane and membrane-bound organelle (Fig. 5). Among biological processes, the highest number of unigenes belonged to cellular metabolic process, and the top three classes of genes were primary metabolic process, macromolecular metabolic process and response to stimulus. Additionally, the molecular function and cellular metabolic process terms contained the highest numbers of unigenes, followed by metabolic process, macromolecular metabolic process and response to stimulus. The GO functional enrichment analysis and unigene expression abundance data will provide useful information for the identification of genes involved in AR development in tetraploid black locust.

### Genes involved in cysteine and methionine metabolism were significantly regulated by IBA during AR formation

We further examined the genes encoding proteins involved in Cysteine and methionine metabolism during the process of adventitious rooting. We identified a number of unigenes (fold change > 2), including 12 methionine-related genes, and 12 ethylene-related genes, associated with the KEGG pathway Cysteine and methionine metabolism (Additional file 11). Among those 62 genes, 60 were identified as auxin-related. The genes down-regulated at stage II included a threonine synthase gene and four S-adenosylmethionine decarboxylase genes; between stages II and IC, the down-regulated genes included six S-adenosylmethionine synthetase-like genes and an aspartate kinase-like gene. Compared with stages II and IC, a total of 21 genes, including 11 up-regulated and 10 down-regulated, were identified as differentially regulated at stages IRP and IAR. The up-regulated genes were primarily members of the S-adenosylmethionine synthetase family, while the down-regulated genes were mostly members of the adenosylhomocysteinase family. S-adenosylmethionine decarboxylase has been known to function as an auxin carrier complex in cellular auxin efflux and influx [44]. These results indicate that most of the genes related to methionine metabolism were significantly up-regulated by IBA treatment. The up-regulation of aspartate aminotransferase and the DNA methyltransferase during stages II and IC suggests that auxin transport occurs in these stages. In other studies, the expression levels of spermidine synthase and S-adenosylmethionine decarboxylase, which are essential for AR formation [45], were up-regulated by IBA treatment. Among the ethylene-related genes, 17 DEGs were identified at stage IRP, with 15 up-regulated and two down-regulated, and 11 DEGs were identified at stage IAR, with eight up-regulated and three down-regulated. Compared with stages II and IC, stages IRP and IAR showed a total of 20 DEGs, with 9 up-regulated and 11 down-regulated.

### The DEGs in each developmental stage in the CK and IBA treatments

To further understand the roles of the DEGs in each developmental stage in the CK and IBA-treated cuttings, we further analyzed the expression levels of a total of 25 genes that showed differential expression in the samples. Among these, several genes were differentially expressed in the AR formation phase, with higher expression in IBA-treated cuttings than in CK, including ACC oxidase 1 (ACO1). Interestingly, the expression of these genes is induced by exogenous auxin in the rooting-competent cuttings of two distantly related forest species, and there are some similar reports that ACO genes lead to AR formation in mung bean and tomato [46, 47]. It appears that IBA-induced ethylene production may contribute to the stimulation of adventitious rooting [47]. Arabinogalactan protein 22 (AGP22) and other AGPs are extracellular proteoglycans that are implicated in many plant growth and developmental processes; for example, AtAGP30 is a non-classical AGP core protein from *Arabidopsis* that is expressed only in roots [48]. Laskowski et al. report that FQR1 is a novel primary auxin-response gene that encodes a flavin nucleotide-binding flavodoxin-like quinone reductase, and accumulation of FQR1 mRNA can be detected in roots and root cultures [49]. AtMRP5, as a newly identified member of the ABC transporter superfamily, controls root development in *Arabidopsis thaliana*, according to Gaedeke et al. [50]*.* Further, Zhang et al. and To et al. have also reported that the genes *ARR8/ARR9* are expressed in the root and that they act partially redundantly to negatively regulate the response of roots to exogenous cytokinin during root meristem initiation [51, 52]. Plants possess a superfamily of arm-repeat proteins, which were reported to be involved in abscisic acid signaling by Kim et al. [53] and in lateral root development by Coates [54].

The DEGs detected encompassed ten gene families. Among the auxin-responsive factor (ARF) family proteins, ARF2 and ARF5 were up-regulated both in CI compared with II and in CAR compared with IAR. Two auxin-responsive factor genes have been found to be involved in auxin signaling and to regulate adventitious rooting [55]. Zinc finger family proteins, specifically of the basic leucine zipper and C2H2 types, were up-regulated in CI compared with II and in CRP compared with IRP. Similar reports have indicated that basic leucine zipper genes promote the modulation of meristems and primordial development. The two wound-responsive family genes and two WRKY transcription factor family genes were significantly down-regulated in CAR compared with IAR and were significantly regulated by IBA. However, a conflicting report by Li et al. indicated that zinc finger proteins and WRKY were down-regulated by IBA treatment during the early stages of adventitious rooting [47]. These results indicate that these differentially expressed genes were directly or indirectly regulated by IBA.

## Conclusion

Transcriptome sequence data for CK and IBA-treated tetraploid black locust cuttings at four developmental stages were obtained using the Illumina sequencing method, with subsequent *de novo* assembly. Despite the economic importance of this tree, the tetraploid black locust genome is not publicly available, and sequence data for tetraploid black locust are limited. Our study generated the first large-scale transcriptome dataset of tetraploid black locust for CK and IBA-treated cuttings. Additionally, the types and quantities of the genes expressed in CK and IBA-treated tetraploid black locust cuttings, along with their functions, classifications, and metabolic pathways, were revealed for the first time. In total, 10,181 and 14,924 DEGs were detected in CK and IBA-treated cuttings, respectively. Our study presents a global view of transcriptome dynamics and differential gene expression analysis for IBA-treated tetraploid black locust cuttings over four developmental stages and provides new insights into the fundamental mechanisms associated with auxin-induced adventitious rooting. Our data constitute a valuable resource for genomic investigations of AR formation in tetraploid black locust cuttings for improving rooting of difficult-to-root varieties.

## Methods

### Plant materials and IBA-treated adventitious root development

Cuttings of tetraploid black locust (*Robinia pseudoacacia* L.) were collected from a 1-year-old seed bearer at the nursery of Northwest Agriculture and Forestry University, Yangling, China. The preparation, experimental plan and IBA-treated cuttings were performed according to the methods described by Quan et al. [18, 19]. Sub-terminal parts of stems 15 cm in length and 10–12 mm in diameter were collected from the same cultivar. The basal 2.5 cm of each cutting was then placed for 4 h in water for the CK or in 5.4 mM IBA as the auxin treatment. The cuttings of tetraploid black locust were subsequently placed on a bench in a glasshouse, and 5 cm portions of the basal parts of cuttings were buried in sand. Cuttings were managed and randomly selected according to the method described by Quan et al. [20]. The 2-cm-long basal stem regions of each cutting, where ARs develop, were cut and collected separately at 0, 15, 20, and 25 days after planting, and the harvested samples were designated control cuttings sampled at stages CI, CC, CRP and CAR for the CK and at stages II, IC, IRP and IAR for the IBA-treated cuttings. Ten cuttings randomly selected from each group were pooled. For each treatment and time point, three biological replicates, each consisting of cuttings from ten base stems, were collected, frozen immediately in liquid nitrogen, and stored at −80 °C prior to RNA extraction.

### RNA separation, cDNA library preparation and Illumina sequencing

Using the manufacturer’s protocol (TIANGEN Biotech, Beijing), the RNA prep Pure Plant Kit was applied to isolate total RNA from each sample. To prevent contamination with genomic DNA, RNA samples were processed with RNase-free DNase I (Takara, Japan). We used an Agilent 2100 Bioanalyzer (Agilent Technologies, Palo Alto, CA, USA) to obtain and quantify RNAs. Thereafter, we used ethidium bromide staining and denatured agarose gel electrophoresis, to inspect the completeness of RNAs. Subsequently, the analysis only included RNA integrity numbers ≥7.5, RNA 28S:18S ratios higher than one and RNA samples with A260/A280 ratios between 1.9 and 2.10.

Based on the manufacturer’s guidelines, Dynal oligo (dT)_25_ beads were used to separate poly(A)^+^ RNA from total RNA of tetraploid black locust in order to conduct Illumina sequencing. We purified the poly (A)^+^ RNA and then cleaved the mRNA into short pieces in a cleavage buffer. Using the random hexamer primer N6, SuperScript III reverse transcriptase and short pieces as templates, we synthesized the first-strand cDNAs. Next, RNase H, buffer, DNA polymerase I and dNTPs were used for second-strand cDNA synthesis. End repair of the double-stranded cDNAs was performed using T4 polynucleotide kinase, T4 DNA polymerase and DNA polymerase I Klenow fragment. Then, T4 DNA ligase was used to ligate the fragments to adapters. The QiaQuick PCR extraction kit was used to isolate the ligated fragments (200 ± 25 bp), which were then eluted in EB buffer and analyzed using agarose gel electrophoresis. Then, we chose appropriate segments as samples for PCR amplification. Eventually, we used an Illumina HiSeq™ 2000 to sequence the library at LC Sciences in Hangzhou, China. All technical steps were performed twice.

### De novo assembly and functional annotation

We discarded empty reads, adapter sequences, and poor sequences from the clean raw reads (with unknown sequences (N) or sequences less than 25 bp). Next, short reads were processed in triplicate for *de novo* assembly of transcriptomes. Then, the triplicate samples were used to collect clean reads into non-repeating transcripts. To enhance the quality of the reads, we also deleted short sequences (<200 bp). Using the Kyoto Encyclopedia of Genes and Genomes (KEGGs) database with an E-value cut-off of 10^−5^ and the Clusters of Orthologous Groups of proteins (COGs), Swiss-Prot, Pfam and NCBI non-redundant protein (Nr) databases, we identified the most important sequences through BLAST searches and annotation. We used Blast2go software to perform functional annotation of Gene Ontology (GO) (www.geneontology.org).

### Identifying differentially expressed genes

We applied RPKM (reads per kilobase per million reads), as the standard value of gene expression [56], to analyze differential gene expression. Using the IDEG6 web tool (http://telethon.bio.unipd.it/bioinfo/IDEG6_form/) [57], we performed statistical comparisons between the CK samples and the IBA-treated samples in terms of RPKM values. In many experiments, the importance of changes in gene expression was assessed based on the *P*-value, limiting the false discovery rate (FDR) at 0.01. In comparisons between libraries, genes showing fold-change >2 with *P* ≤0.05 were defined as differentially expressed. For up- and down-regulated unigenes, we used WEGO to compare their GO classifications [33].

### Real-time quantitative PCR (RT-qPCR)

For RT-qPCR, we chose 21 candidate genes (Additional file 12) that showed differential expression to verify the Illumina sequencing data. Reverse transcription was performed using the PrimeScript RT reagent kit (Takara, Dalian, China) and 1 μg of RNA. The products of reverse transcription were diluted to 150 ng/μl in double-distilled water. Primer Premier 6.0 was used to generate antisense and sense primers. Using TaKaRa SYBR Premix Ex Taq II (Perfect Real Time), we performed RT-qPCR in a 20 μl reaction volume with a RC96 Real-Time PCR detection system (Rocipe Laboratories, Hercules, CA, USA). The 18S rRNA gene was used as the endogenous reference gene. After 2 min of predenaturation at 94 °C to measure fluorescence, RT-qPCR was performed with forty-five cycles of denaturation at 94 °C for 10, annealing at 60 °C for 15 s and elongation at 72 °C for 30 s. To ensure the absence of primer dimers, the melting curve was analyzed in the first cycle. The 2^-△△Ct^ method [14] was used to confirm the relative expression of target genes. Each sample was analyzed in triplicate. We used Origin (Version 8.0) to analyze the data and then log-transformed all data obtained from the q-PCR analysis.

## Additional files

Additional file 1: ength distribution of assembled uni-transcripts and uni-genes. (DOCX 14 kb)
Additional file 2: Summary of the blastx results for the tetraploid black locust transcriptome against five databases. (DOCX 13 kb)
Additional file 3: RNA-seq-based transcriptome dynamics of CK cuttings during AR development. (DOCX 561 kb)
Additional file 4: The expression levels of genes that increased continuously in both IBA-treated and CK cuttings. (XLSX 10 kb)
Additional file 5: Venn diagrams of unigenes showing differential expression in pairwise comparisons of developmental stages in the IBA treatment. (DOCX 93 kb)
Additional file 6: Gene Ontology classification of the differentially expressed genes of IBA treated from stage II to stage IAR. (DOCX 2985 kb)
Additional file 7: Genes differentially expressed between IBA-treated and CK cuttings at each developmental stage. (DOCX 201 kb)
Additional file 8: Gene Ontology classifications of the differentially expressed genes in CK and IBA from all four stages. (DOCX 4032 kb)
Additional file 9: Genes involved in major processes associated with IBA-treated AR developmental stage. (XLSX 12 kb)
Additional file 10: List of most significantly up- and down-regulated GO classifications. (XLSX 63 kb)
Additional file 11: List of most significantly up- and down-regulated KEGGs. (XLSX 4500 kb)
Additional file 12: List of the genes and primers selected for q-PCR validation. (DOCX 15 kb)
