# Supplementary material for: De novo sequencing and comparative transcriptome analysis of adventitious root development induced by exogenous indole-3-butyric acid in cuttings of tetraploid black locust
Source: BMC Genomics. 2017 Feb 16;18:179. doi: 10.1186/s12864-017-3554-4 (PMC5314683; doi:10.1186/s12864-017-3554-4)
Supplement: Additional file 1: — ength distribution of assembled uni-transcripts and uni-genes. (DOCX 14 kb) [file 12864_2017_3554_MOESM1_ESM.docx]

**Additional file 1. Length distribution of assembled uni-transcripts and uni-genes**.

| Classification |  | Number bp | |  | Percentage % | |
| --- | --- | --- | --- | --- | --- | --- |
| Length |  | Uni-transcripts | Uni-genes |  | Uni-transcripts | Uni-genes |
| 200-300 |  | 35678 | 28954 |  | 35.14 | 37.17 |
| 300–600 |  | 23897 | 25772 |  | 20.28 | 28.14 |
| 600–1000 |  | 24562 | 15784 |  | 15.28 | 13.13 |
| 1000–1600 |  | 18767 | 10567 |  | 13.12 | 10.21 |
| 1600–2000 |  | 8953 | 38034 |  | 4.12 | 3.15 |
| >2000 |  | 16522 | 9985 |  | 12.06 | 8.21 |
| Total numbers |  | 127,038 | 101,209 |  | 100.00 | 100.00 |
| Average length |  | 986 | 852 |  |  |  |
| Total length |  | 125353356 | 86239985 |  |  |  |
| N50 length |  | 1643 | 1449 |  |  |  |
