# Supplementary material for: De novo sequencing and comparative transcriptome analysis of adventitious root development induced by exogenous indole-3-butyric acid in cuttings of tetraploid black locust
Source: BMC Genomics. 2017 Feb 16;18:179. doi: 10.1186/s12864-017-3554-4 (PMC5314683; doi:10.1186/s12864-017-3554-4)
Supplement: Additional file 2: — Summary of the blastx results for the tetraploid black locust transcriptome against five databases. (DOCX 13 kb) [file 12864_2017_3554_MOESM2_ESM.docx]

| Annotation batabase | Annotation number | Annotation percents (%) |
| --- | --- | --- |
| Nr annotation | 75260 | 74.36 |
| KOGs annotation | 39822 | 39.35 |
| KEGG annotation | 68351 | 67.53 |
| Pfam annotation | 58459 | 57.76 |
| Swiss-Prot annotation | 43558 | 43.04 |
| All annotation | 101209 | 100.00 |

**Additional file 2 Summary of the blastx results for the tetraploid black locust transcriptome against five databases**.
