# Supplementary material for: De novo sequencing and comparative transcriptome analysis of adventitious root development induced by exogenous indole-3-butyric acid in cuttings of tetraploid black locust
Source: BMC Genomics. 2017 Feb 16;18:179. doi: 10.1186/s12864-017-3554-4 (PMC5314683; doi:10.1186/s12864-017-3554-4)
Supplement: Additional file 3: — RNA-seq-based transcriptome dynamics of CK cuttings during AR development. (DOCX 561 kb) [file 12864_2017_3554_MOESM3_ESM.docx]

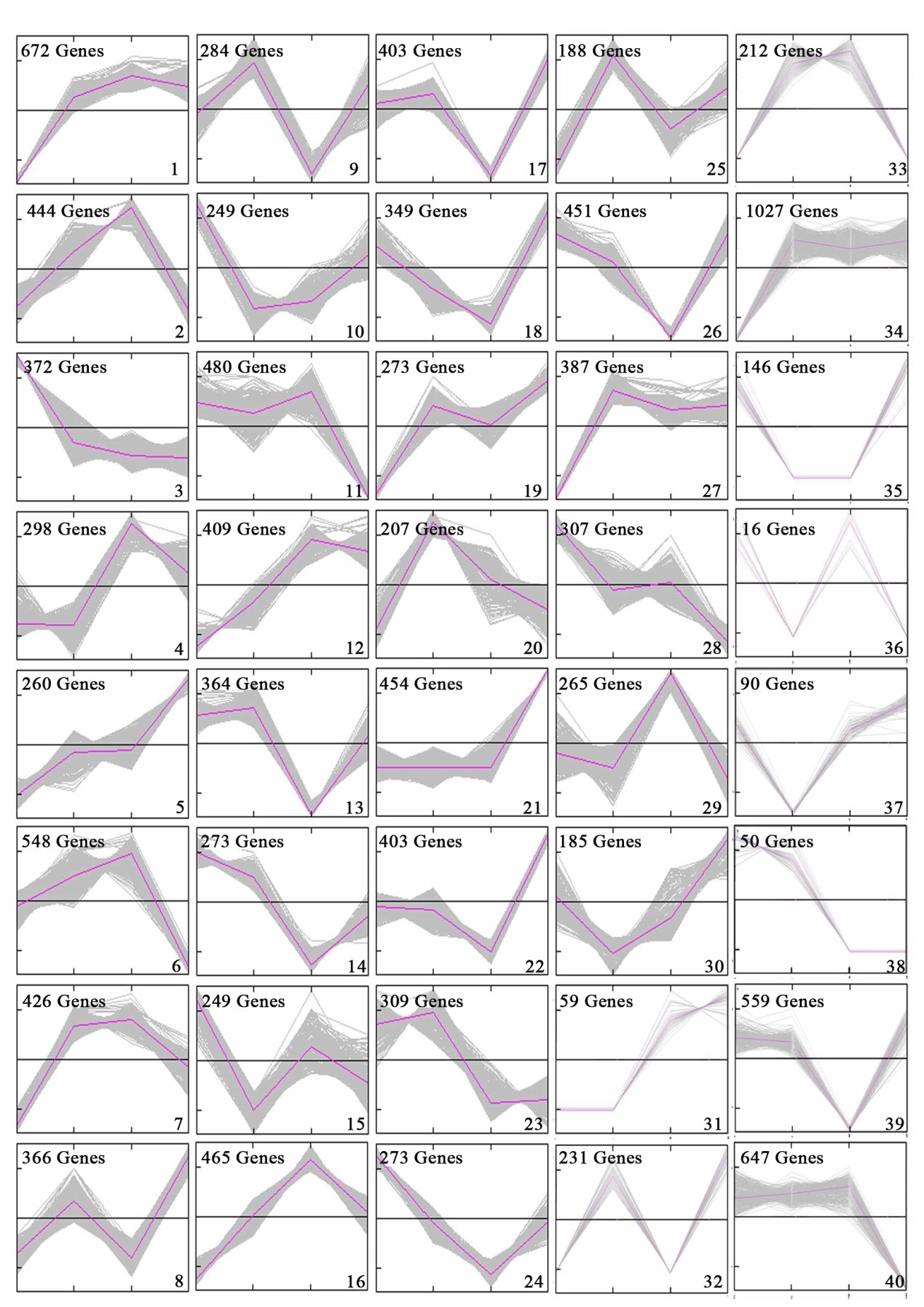


Additional file 3 RNA-seq-based transcriptome dynamics of CK cuttings during AR development.

The fold-change >2.0 for each gene was used for the hierarchical clustering analysis at each of the four selected developmental stages (II, IC, IRP and IAR). The 10,181 genes were classified into 40 regulation patterns (groups 1-10, 14-18, and 20-24).
