# Supplementary material for: De novo sequencing and comparative transcriptome analysis of adventitious root development induced by exogenous indole-3-butyric acid in cuttings of tetraploid black locust
Source: BMC Genomics. 2017 Feb 16;18:179. doi: 10.1186/s12864-017-3554-4 (PMC5314683; doi:10.1186/s12864-017-3554-4)
Supplement: Additional file 5: — Venn diagrams of unigenes showing differential expression in pairwise comparisons of developmental stages in the IBA treatment. (DOCX 93 kb) [file 12864_2017_3554_MOESM5_ESM.docx]

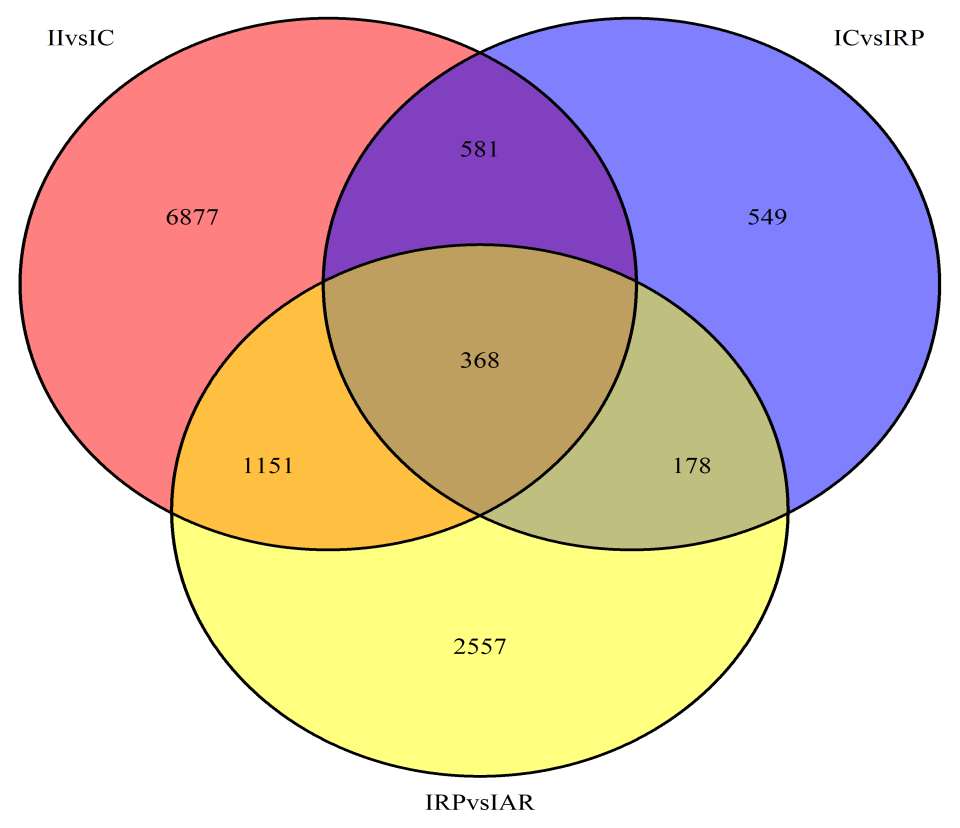


Additional file 5 Venn diagrams of unigenes showing differential expression in pairwise comparisons of developmental stages in the IBA treatment.
