# Supplementary material for: De novo sequencing and comparative transcriptome analysis of adventitious root development induced by exogenous indole-3-butyric acid in cuttings of tetraploid black locust
Source: BMC Genomics. 2017 Feb 16;18:179. doi: 10.1186/s12864-017-3554-4 (PMC5314683; doi:10.1186/s12864-017-3554-4)
Supplement: Additional file 6: — Gene Ontology classification of the differentially expressed genes of IBA treated from stage II to stage IAR. (DOCX 2985 kb) [file 12864_2017_3554_MOESM6_ESM.docx]

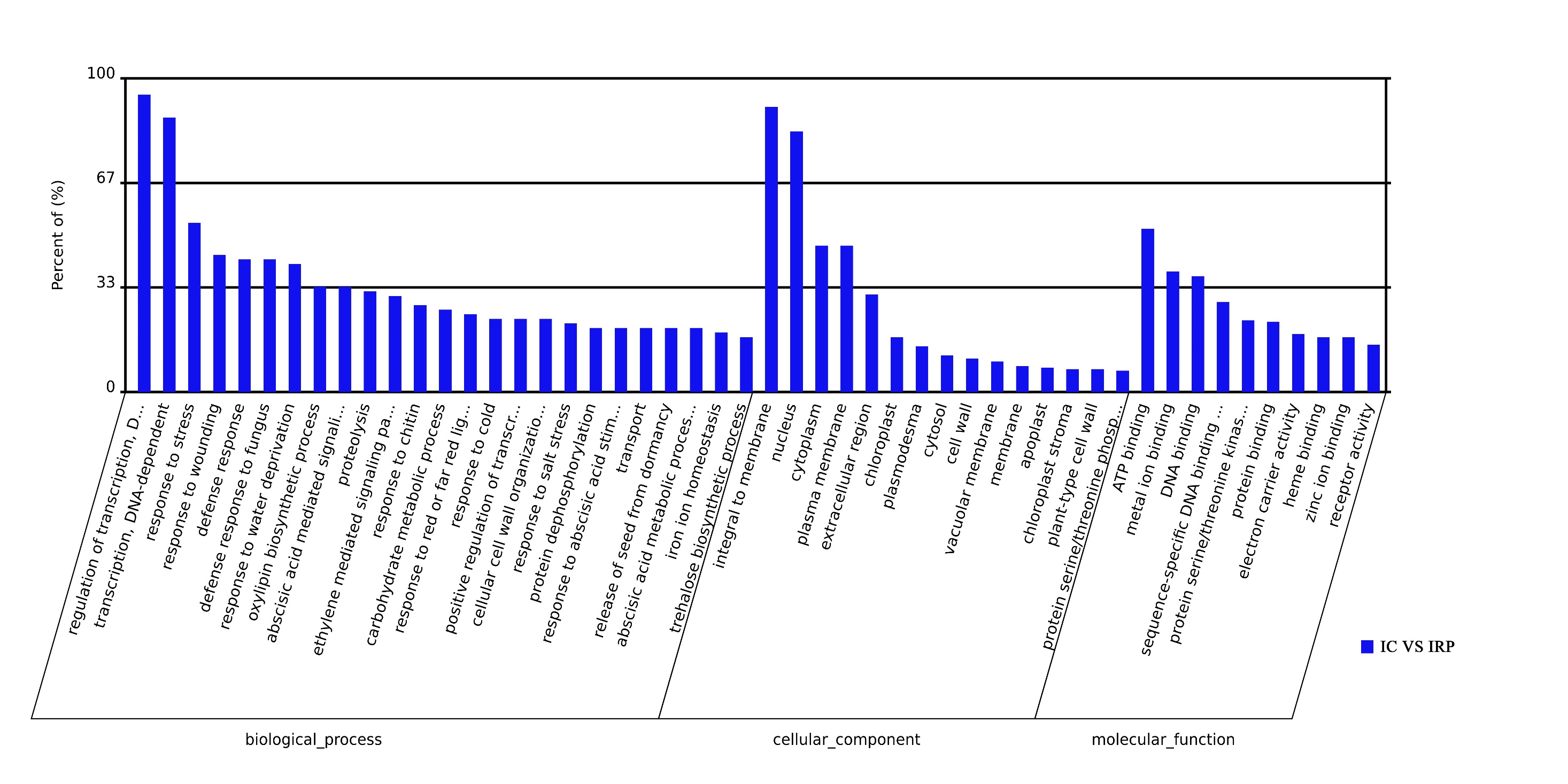

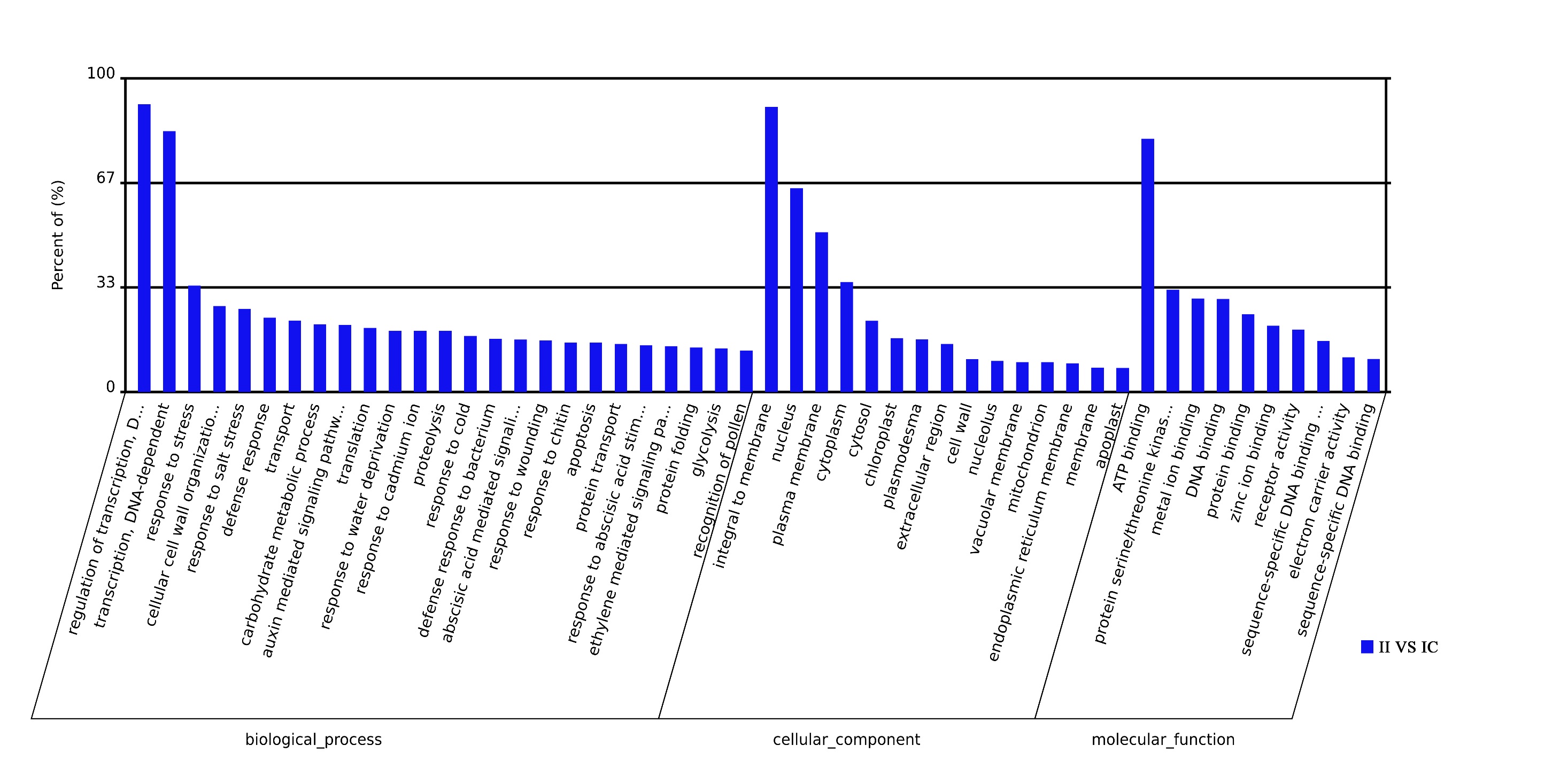

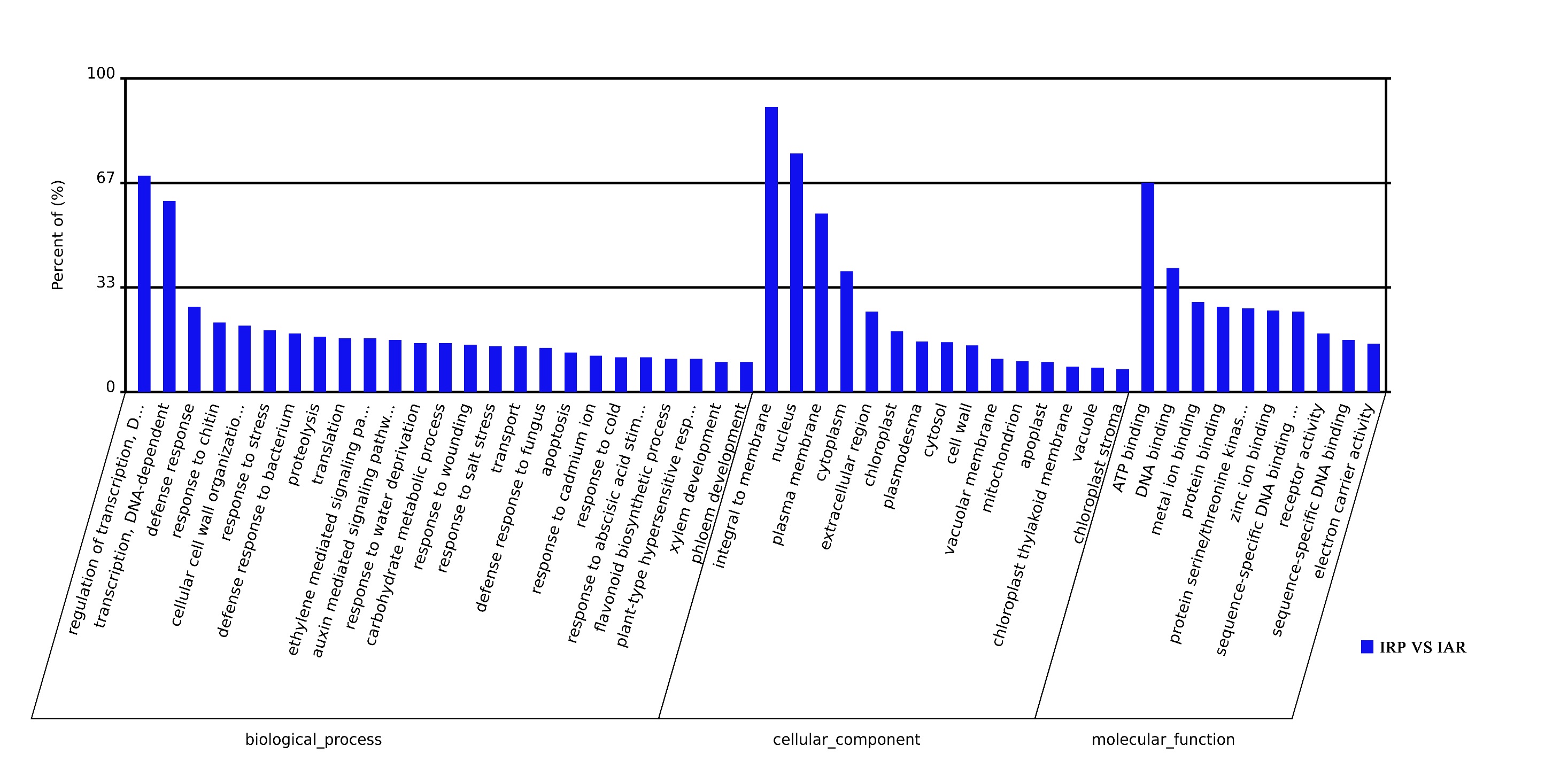


Additional file 6 Gene Ontology classification of the differentially expressed genes of IBA treated from stage II to stage IAR.
