# Supplementary material for: De novo sequencing and comparative transcriptome analysis of adventitious root development induced by exogenous indole-3-butyric acid in cuttings of tetraploid black locust
Source: BMC Genomics. 2017 Feb 16;18:179. doi: 10.1186/s12864-017-3554-4 (PMC5314683; doi:10.1186/s12864-017-3554-4)

**Additional file 8 Gene Ontology classifications of the differentially expressed genes in CK and IBA from all four stages.**


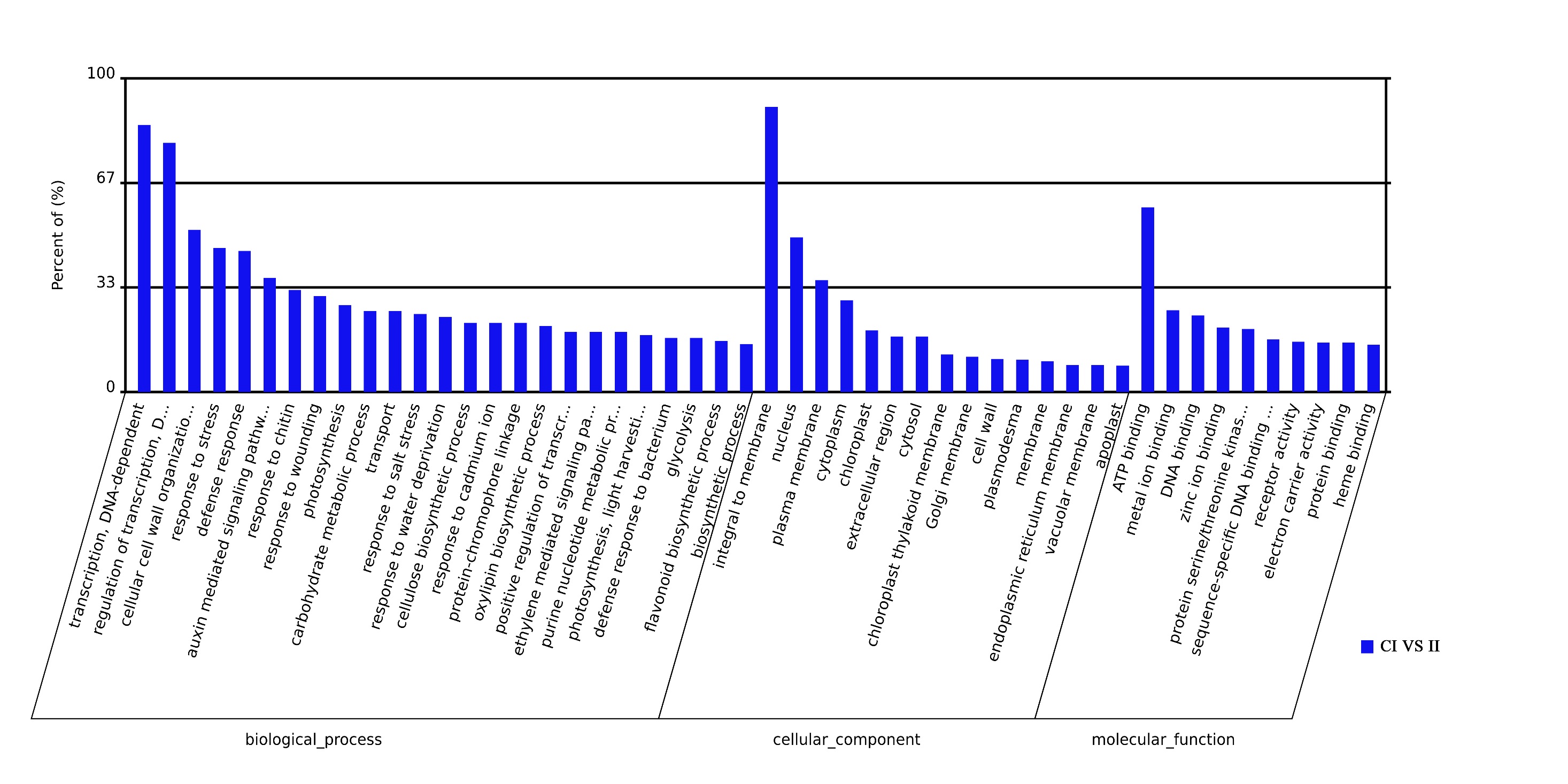

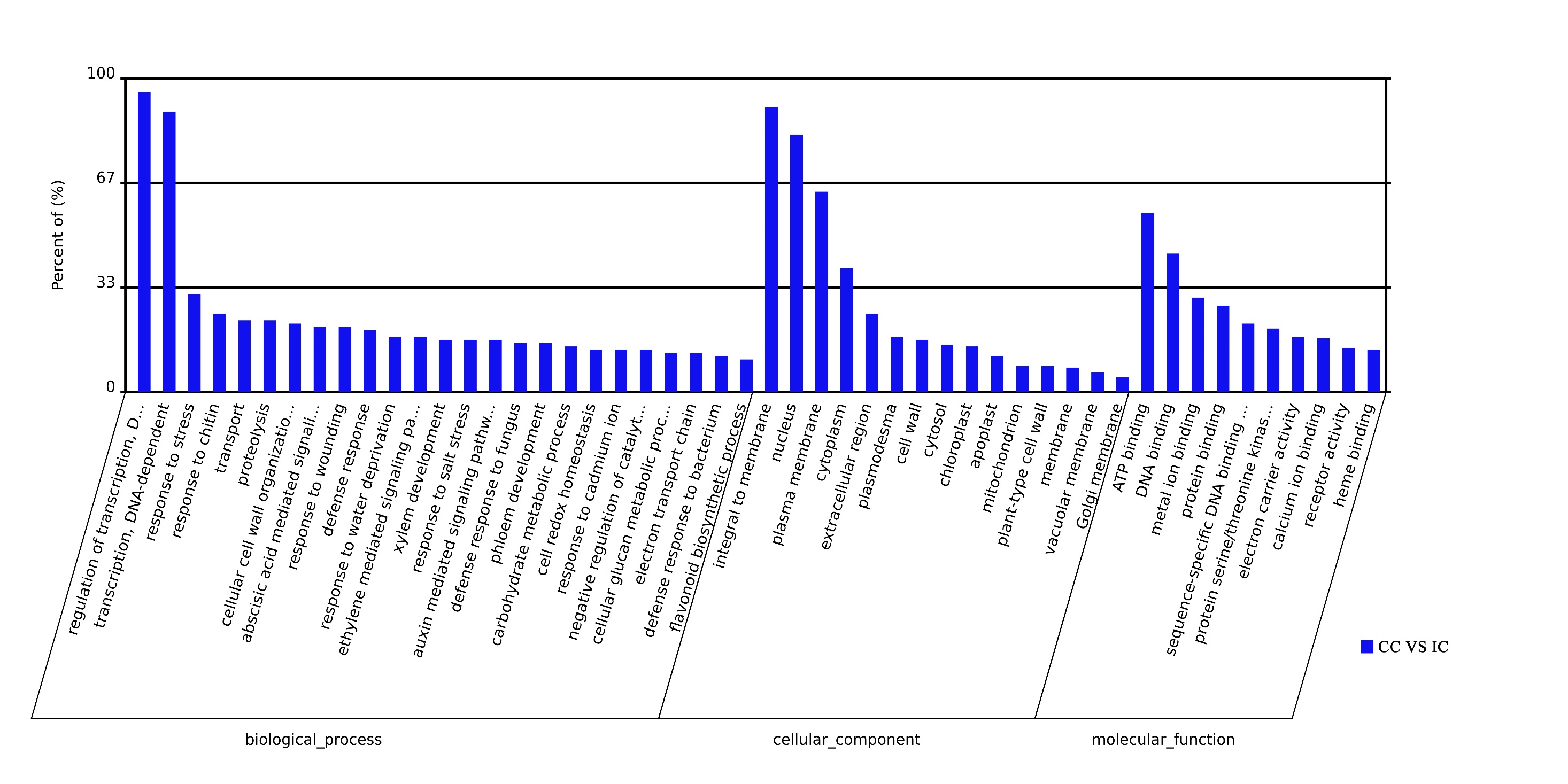

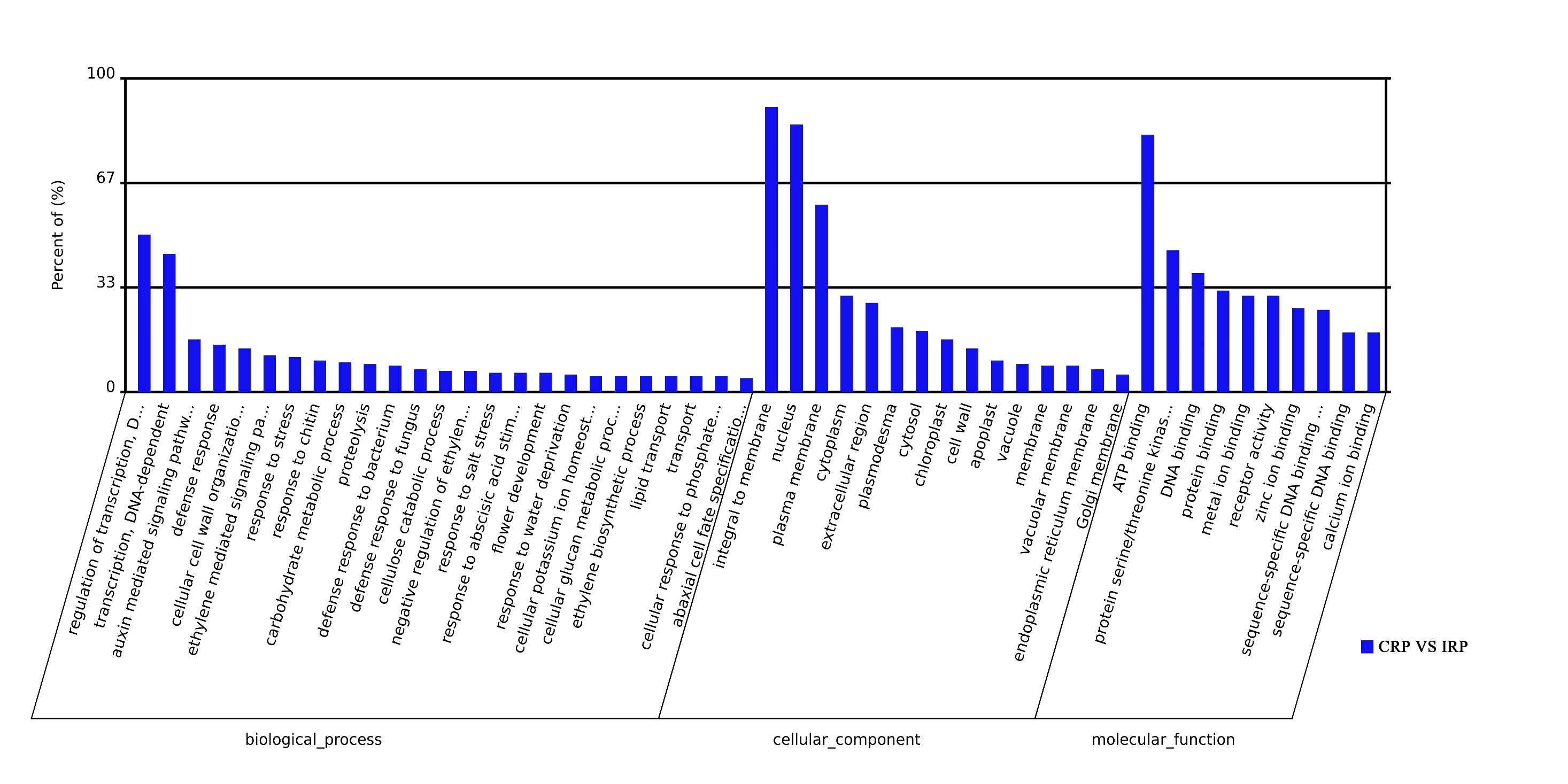

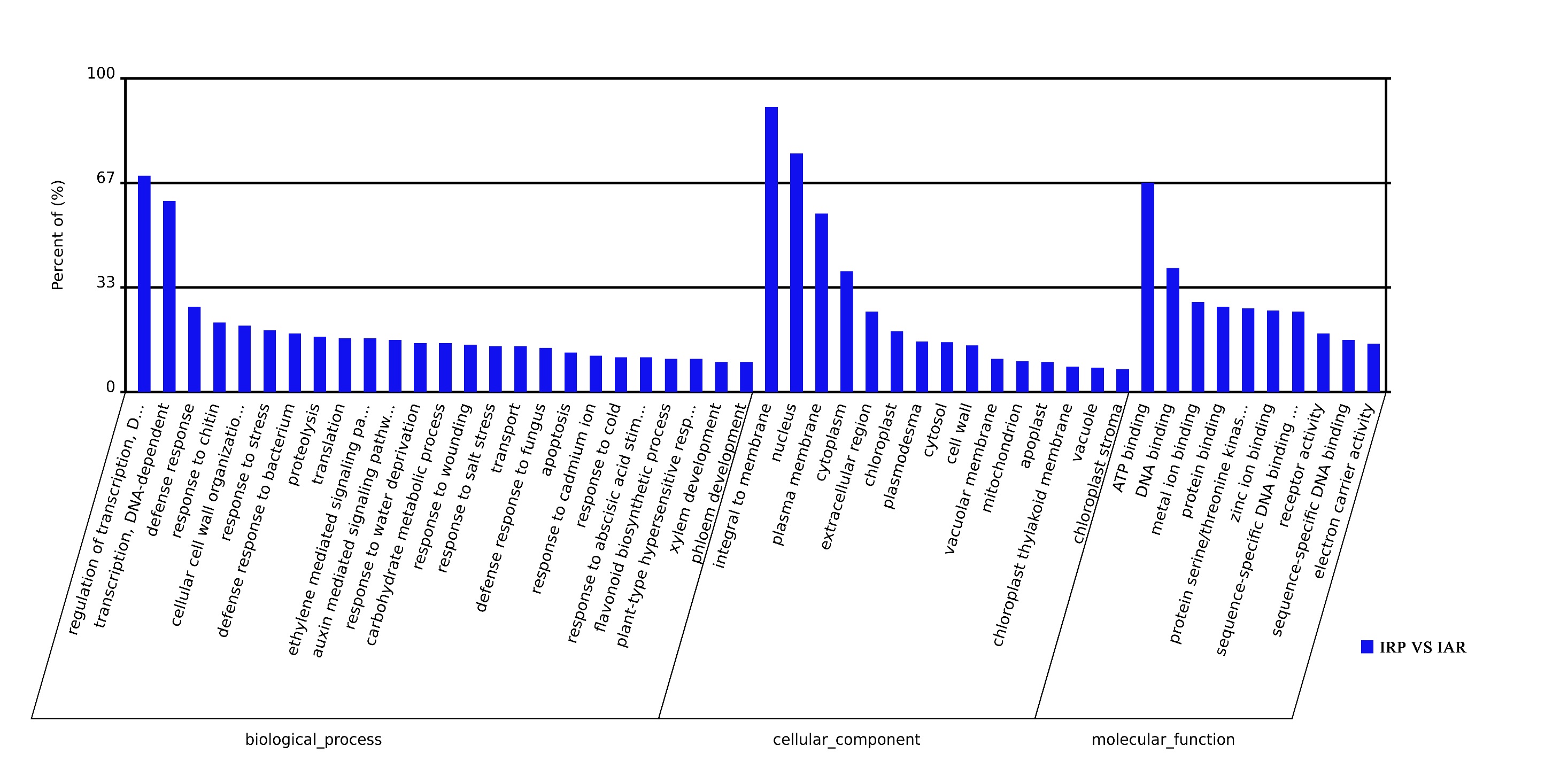

Supplement: Additional file 8: — Gene Ontology classifications of the differentially expressed genes in CK and IBA from all four stages. (DOCX 4032 kb) [file 12864_2017_3554_MOESM8_ESM.docx]
