# Supplementary material for: De novo sequencing and comparative transcriptome analysis of adventitious root development induced by exogenous indole-3-butyric acid in cuttings of tetraploid black locust
Source: BMC Genomics. 2017 Feb 16;18:179. doi: 10.1186/s12864-017-3554-4 (PMC5314683; doi:10.1186/s12864-017-3554-4)
Supplement: Additional file 12: — List of the genes and primers selected for q-PCR validation. (DOCX 15 kb) [file 12864_2017_3554_MOESM12_ESM.docx]

Additional file 12 List of the genes and primers selected for q-PCR validation.

| Unigene id | Gene name | Forward primer sequence (5'–3') | Reverse primer sequence (5'–3') |
| --- | --- | --- | --- |
| Trb67341 | Delta-l- pyrroline-5-carboxylate synthetase | CCTTGTTCAGATAGCGGCATT | TGACCTTGTGTAAGACAGCATT |
| Trb24356 | auxin-repressed protein | TGACTCCAACGACACCAACA | TCTCCACTGTAGAGCCAATCAT |
| Trb62538 | ethylene responsive transcription factor | AAGGGTGAAGGAAAGAAGAGTG | AAGGTGCCAAGCCAGACA |
| Trb65012 | transcription factor BHLH | AGCCAAGCCAATCACCTGAA | TCTTAGCATGGAGAGCCTGTC |
| Trb76022 | wound induced protein | TAGGCGGCAGTAGCATAGC | TGGACTTGGATGTGAATGTGTT |
| Trb59815 | 2,4-Dinducible glutathione S-transferase | GCCGTTATGCTTCAACCTACT | GAGAGTGAGTGCCCAAAGTTC |
| Trb70207 | l-aminocyclopropane-l-carboxylate oxidase | CCAGGTTGTGCTTCATCATCC | CGCAGCCGAATAAGAAGTTGT |
| Trb58732 | heat shock cognate protein | CGATCTAGGCACCTGCTACT | GTGTCAGTGAAGGCAACATAAG |
| Trb61918 | zinc finger protein | TTGTTGGTGCTGCTGTTGTT | GCCAGTCATCGCAAGTCTTC |
| Trb64635 | phloem protein | GCAAGAGCACTCTCAATCACC | TCAGTTCAGCCACCTCAGTC |
| Trb62764 | WRKY transcription factor | CAGCCATGTGAGCGGTAACTA | CCATTCCTCCACCAACACCAT |
| Trb63108 | Auxin induced in root cultures protein | GGTCCAGTGAAGGTAAGATGTC | GCTTGTGTTGGCAATCTATGTT |
| Trb80229 | Auxin-responsive protein IAA | CAACTGATGAACAATCCGATGA | GATGAGGAAGGTGAAAGCAATG |
| Trb58447 | Cytochrome P450 | AGGAGGAGACTATCAACGACAT | TGGAAGCCTTGTGTTCATTCA |
| Trb55666 | Flowering promoting factor protein | CTGGAGCTGAGGCATACGA | TCAAGCACCGCATAGGAAGT |
| Trb67916 | Peroxidase | AAGCACGGAACAATCGGAAC | ACACCTTCAATGCCAACTTCG |
| Trb62373 | Beta-D-xylosidase | CGGATTGTGTTGCGAAGTAGT | AAGCGTGCGTGTTGGAAG |
| Trb67209 | Serine/threonine kinase | TCTCTGCGACTTCTTCTTCAGT | TCCGTGACTTCCACCTCCT |
| Trb76511 | S-adenosyl-L-methionnine decarboxylase | ACTCTGCCTCTGCTGATTCTGT | CACGGCTGCTGAACCTGTCT |
| Trb63012 | S-adenosyl-L-methionnine synthetase | TCCTCATGGTGATGCTGGTCTC | AGCAAGTCCACTGGCAACAATG |
| 18S |  | TAGTTGGTGGAGCGATTTGTC | CAGAACATCTAAGGGCATCACAG |
